# Biological Impact of Extrusion Bioprinting Nasoseptal Chondrocytes for Tissue Engineering Applications

**DOI:** 10.3390/jfb17040163

**Published:** 2026-04-01

**Authors:** Thomas Harry Jovic, Josh Roberts, Feihu Zhao, Shareen Heather Doak, Iain Stuart Whitaker

**Affiliations:** 1 Reconstructive Surgery & Regenerative Medicine Research Centre, Swansea University, Swansea SA2 8PP, UK; 2 Welsh Centre for Burns & Plastic Surgery, Morriston Hospital, Swansea SA6 6NL, UK; 3 Department of Biomedical Engineering & Zienkiewicz Institute, Faculty of Science and Engineering, Swansea University, Swansea SA1 8EN, UKfeihu.zhao@swansea.ac.uk (F.Z.); 4 Swansea University Medical School, Swansea University, Swansea SA2 8PP, UK

**Keywords:** cartilage, bioprinting, shear stress, computational fluid dynamics

## Abstract

Shear stress is a significant consideration in 3D bioprinting systems, with implications for cell viability and behaviour. This study hypothesised that relevant levels of shear stress would be generated during the process of 3D bioprinting human nasoseptal chondrocytes in a nanocellulose alginate bioink, with implications for cell viability and chondrogenic gene expression. Through a combined approach of in silico modelling and in vitro testing, we assessed chondrocyte viability and gene expression immediately within the first 72 h post-printing. Cell viability was determined using live–dead, alamarBlue and lactate dehydrogenase assays immediately and 24 h post-printing compared to cell-only and unprinted cell–biomaterial controls. Gene expression analysis of Type 2 collagen, SOX9, aggrecan and alkaline phosphatase gene expression was performed 4 h and 72 h post-printing. Computational fluid dynamics predicted a shear stress of 292 Pa and maximum fluid velocity of 19 mm/s during the bioprinting process. No statistically significant cell death or cell lysis was detected between groups immediately post-printing; however, statistically significant chondrocyte cell death was observed at 24 h in the printed group (*p* = 0.047). Moreover, the bioprinting process evoked a transient initial rise in both chondrogenic (SOX9, aggrecan) and osteogenic gene expression (ALP) with a marked suppression in type 2 collagen expression at 72 h (0.05, *p* = 0.0005), indicating biological effects evoked by shear stress during printing. This study highlights the importance of optimising the bioprinting process to facilitate low shear stress conditions for durable cartilage tissue engineering.

## 1. Introduction

The clinical translation of 3D bioprinting technology has the potential to yield novel and personalised surgical options for patients [1,2]. The current gold standard approaches to reconstructing soft tissue defects depend largely on the use of autologous tissue transfer to restore form and function [3]. 3D bioprinting has the potential to obviate the need for tissue transplantation and to offer the restoration of form and function whilst lessening the burden of destructive, painful or disfiguring donor sites [4]. Additional advantages of 3D bioprinting in surgery include the potential to replicate the anisotropy of biological tissues through tailoring structure at the macro- to nano-scale in order to optimise ECM composition, cellular interactions and topography. 3D bioprinting could, as such, offer a more reliable, bespoke and less disruptive option for patients.

The biological demands on 3D printing include facilitating processes such as cell adhesion and proliferation in addition to the mechanical roles such as tissue support and durability [5], with extensive research conducted in the field of bioink optimisation [6]. In contrast to 3D printing, the entire process of bioprinting must be deemed biocompatible in order to be a viable in vivo solution. As such, some elements of the 3D bioprinting process must be refined to enable interaction with cellular material, including printing temperatures and mild, non-cytotoxic crosslinking methods to preserve cellular integrity [7].

Extrusion-based bioprinting, or bioplotting, is the most common type of bioprinting, involving the deposition of cells embedded in a bioink through a nozzle driven by pneumatic, piston or screw forces [8,9]. This process may be slower than laser, stereolithography or inkjet bioprinting, but extrusion bioprinting typically has the advantages of tunability, superior cell viability and the potential for complex multi-layered constructs to be derived from multiple nozzles incorporating different materials and cell types [10,11]. The printer usually has a cartridge connected to a straight or conical nozzle in order to enable the precise deposition of the filament, but depending on the viscosity of the bioink, high printing pressures may be warranted to enable continuous extrusion, often at the expense of cell viability [12].

Biocompatibility in 3D bioprinting is a major challenge: the biomaterial, crosslinking agent and printing process each have the potential to impair cellular integrity. Moreover, cells are environmentally responsive, meaning stresses exerted on the cells during the bioprinting process may confer biologically significant effects on cell behaviour. Chondrocytes in particular are known to respond to mechanical stimuli, particularly in articular cartilage, and these stimuli may be converted into a mechanotransduction signal to increase chondrogenesis and/or proliferation which can be exploited in vitro [13,14,15]. The reactions to mechanical cues are detected by deformation of the pericellular matrix and cell surface receptors, such as integrins, channelomes and the primary cilium, leading to the induction of gene expression that directs extracellular matrix production. The nature of the stress may evoke cartilaginous or mineralised matrix as a response highlighting the importance of appropriate biomechanical signals to the chondrocyte and pericellular matrix [16,17]. However, unlike articular cartilage, the biological impact of shear stresses on nasoseptal chondrocytes is less well characterised, in particular with regard to stresses evoked during 3D bioprinting [17].

The process of extrusion 3D bioprinting involves the conversion of extrusion pressure to shear stress [18]. The printer nozzle is one factor that may influence the degree of shear experienced by cells in bioinks. Specific features pertaining to the nozzle geometry, such as shape, length and diameter, can each have an influence on the degree of shear stress in the system, which may additionally affect cell viability [19]. In addition to having an impact on viability, some cells and tissues are mechanosensitive, and even at non-cytotoxic levels of shear, the shear experienced through the extrusion bioprinting process may be sufficient to evoke a cellular response [20]. Naturally, phenotypic retention in the intended tissue type is paramount for 3D bioprinting processes and divergence into unintended lineages may have adverse effects on the quality of the tissue that is produced.

Our hypothesis was that the extrusion bioprinting process would generate biologically significant levels of shear stress, with implications for cell viability and gene expression. Our aim was to combine computational modelling and biological analysis to model and quantify the effect of 3D printing human nasoseptal chondrocytes in a biologically optimised nanocellulose–alginate bioink at the minimum extrusion pressure for effective bioprinting. In order to ascertain this, we derived an in silico approach to predict the shear stress experienced using the 3D bioprinting process and biologically validate this effect using human nasoseptal chondrocytes to assess immediate and delayed gene expression and cell viability post-printing.

## 2. Materials and Methods

### 2.1. Human Nasoseptal Cartilage Cell Isolation and Culture

Human nasoseptal chondrocytes (hNCs) were isolated from excess nasoseptal cartilage acquired from septorhinoplasty procedures with informed patient consent (IRAS ID 99202). Cartilage tissue was diced into 1 mm^3^ pieces and digested in 0.4% pronase for 1 h at 37 °C, 5% CO_2_ with gentle agitation, followed by secondary digestion in 0.2% collagenase type I solution for 18 h. The resultant cell suspension was filtered through a 40 μm cell strainer and centrifuged at 350 g for 6 min. Cells were cultured in 5% CO_2_ at 37 °C with the culture medium changed every 2–3 days. The culture medium comprised Dulbecco’s Modified Eagle Medium without glucose (Sigma-Aldrich, Poole, UK) supplemented with 10% foetal bovine serum (Sigma-Aldrich, Poole, UK), 100 μg mL^−1^ penicillin and 100 U mL^−1^ streptomycin (Sigma-Aldrich, Poole, UK), 1 mM glucose (SigmaAldrich, Poole, UK), and 0.1% non-essential amino acids (Thermo Fisher Scientific, Paisley, UK). Cells were grown to 70–80% confluence and passaged using 0.05% trypsin-EDTA (Thermo Fisher Scientific, Paisley, UK). Cells were acquired from three separate patients and expanded between passages 1 to 4 for use in these experiments.

### 2.2. Bioink Formulation

Nanocellulose particles were produced from raw wood chip biomass using AVAP technology, which fractionates the biomass into cellulose, lignin and hemicelluloses using ethanol and sulphur dioxide. The resultant nanocellulose blend formulation, BioPlus Nanocellulose Blend (GranBio, Atlanta, GA, USA), was mixed with 2.5% *w*/*v* sodium alginate (Sigma Aldrich, St. Louis, MO, USA) in a 75:25 volume ratio to produce a nanocellulose–alginate (NCA) bioink capable of crosslinking with 0.5 mM calcium chloride solution as previously reported, yielding an elastic modulus of 52.6 kPa [21]. The NCA bioink was mixed with hNCs at a seeding density of 3 × 10^6^ cells per mL. We have previously validated the rheological properties of the bioink which is known to exhibit shear thinning properties and a G′/G″ indicating a dominance of elastic behaviour for post-printing shape fidelity [21,22].

### 2.3. Extrusion-Based 3D Printing

The cell-laden NCA bioink was extruded through a 22 G nozzle through the use of a CELLINK INKredible pneumatic extrusion bioprinter (CELLINK, Gothenburg, Sweden). This nozzle size was used in line with our previously published studies on print fidelity and resolution with this bioink [21,22]. Prior to commencing 3D printing, the minimal extrusion pressure was determined through increasing the pneumatic pressure to the point that material was extruded from the nozzle in a continuous filament. This value of 30 kPa was determined at the minimum extrusion pressure and thereafter set as the printing pressure for the cell-laden bioink.

Stereolithography files (.stl) of hemispheres containing 100 μL of bioink were designed using the Microsoft 3D Builder app and converted to G-code for the INKredible printer using CELLINK Heartware (Version 2.4, CELLINK, Gothenburg, Sweden) software to provide layer-by-layer printing instructions.

As a control to the bioprinting process, 100 μL hemispheres (7.26 mm diameter) of the same cell-laden bioink material were produced using a 1 mL syringe with no nozzle and extruded gently by hand.

Hemispheres were produced in triplicate for each condition and immersed in 100 μL of 0.5 M calcium chloride solution for 5 min until crosslinking was achieved (Supplementary Figure S1). The resultant hemispheres were washed three times with warm phosphate-buffered saline (PBS) and placed into the wells of a 48-well plate with 750 μL of culture medium. The constructs were maintained at 37 °C and 5% CO_2_ until analysed.

### 2.4. AlamarBlue Cell Viability Analysis

The AlamarBlue dye (Thermo Fisher) was used to provide an indication of cell viability and metabolic activity. A 10% (*v*/*v*) AlamarBlue solution was made by adding the solution to the culture medium, which was added to the 3D printed cell-laden biomaterials, unprinted cell-laden biomaterials or cells only for 4 h at 37 °C and 5% CO_2_. To demonstrate the effects on cell viability and metabolic activity, the AlamarBlue assay was performed immediately after printing and repeated at 24 h with alamarBlue media alone used to provide control values. The colorimetric change was quantified using a plate reader (POLARstar Omega spectrophotometer, BMG LABTECH, Ortenberg, Germany) in which absorbency readings were taken at 570 (reduced form) and 600 nm (oxidised form) wavelengths. The subsequent readings were used to calculate the proportion of reacted (reduced) to unreacted (oxidised) AlamarBlue in each condition.

### 2.5. Lactate Dehydrogenase Assay

A lactate dehydrogenase assay (Merck, Darmstadt, Germany) was used to determine immediate cytotoxicity (cell lysis) in the printed and unprinted constructs after 1 h of printing due to direct cellular trauma or osmotic lysis from crosslinking. Cell-laden biomaterials (300,000 cells per pellet) were incubated in standard culture conditions overnight alongside wells containing cell-only controls (300,000 cells per well). Lysis buffer solution was added to three of the cell-only wells and left to incubate for 45 min. A total of 50 μL of media from each well was transferred to a 96-well plate alongside the provided LDH positive control samples. An equal volume of reaction mixture was added to each well for 30 min at room temperature and protected from light. After 30 min, 50 μL of stop solution was added to each well and mixed by tapping. The plate was read at 490 nm and 680 nm absorbance using a plate reader (POLARstar Omega spectrophotometer, BMG LABTECH, Ortenberg, Germany).

### 2.6. Live–Dead Cell Viability Assay

A live–dead mammalian cell viability assay kit (Thermo Fisher Scientific, Waltham, MA, USA) was used to visualise live and dead cells within the printed and unprinted biomaterial constructs. Immediately and 24 h post printing, a solution of PBS containing 1:1000 Calcein-AM dye and 1:500 Ethidium homodimer-1 was produced and protected from light. The solution was added to the wells of interest, and left to incubate for 45 min. The biomaterial-cell hemispheres were visualised using an inverted fluorescent microscope (Olympus, Tokyo, Japan) at 10× magnification. The live and dead cells were visualised using separate fluorescent channels and merged to create a single image. This process was repeated at four different points within the hemisphere. ImageJ software (Version 1.54, National Institute of Health, Bethesda, MD, USA) was used to count the number of live and dead cells in each image, yielding a mean percentage viability for all cells visualised in each condition.

### 2.7. Gene Expression Analysis

#### 2.7.1. RNA Extraction

Cell–biomateral hemispheres were degraded through freezing in a suspension of TRIzol and EDTA. The frozen mixture was homogenised using a TissueRuptor II probe (Qiagen, Hilden, Germany) for 30 s. The mixture was combined with chloroform and centrifuged at 14,500× *g* for 15 min. The supernatant was then processed using the Qiagen RNEasy Mini kits. RNA concentration and quality was determined using a NanoDrop Spectrophotometer (Thermo Fisher)and frozen at −80 °C for storage.

#### 2.7.2. Quantitative Real-Time Polymerase Chain Reaction

Triplicates of each biomaterial were mixed with 500 µL TRIzol (Invitrogen, Thermo Fisher, Carlsbad, CA, USA) and subsequently degraded with a TissueRuptor II probe for 30 s. The lysate was processed using Qiagen QIAshredder and Rneasy Mini Extraction kits (Qiagen) to yield RNA for reverse transcription. The RNA was converted to cDNA through reverse transcription, and quantified for the expression of SOX9, COL2A1, ALP and ACAN relative to housekeeping gene expression (RPL13A). Each material was harvested for RNA extraction and PCR analysis at 4 and 72 h of culture. All relative gene expression values were expressed as fold changes using the ΔΔCt method.

### 2.8. Computational Fluid Dynamics

**Figure 1:**
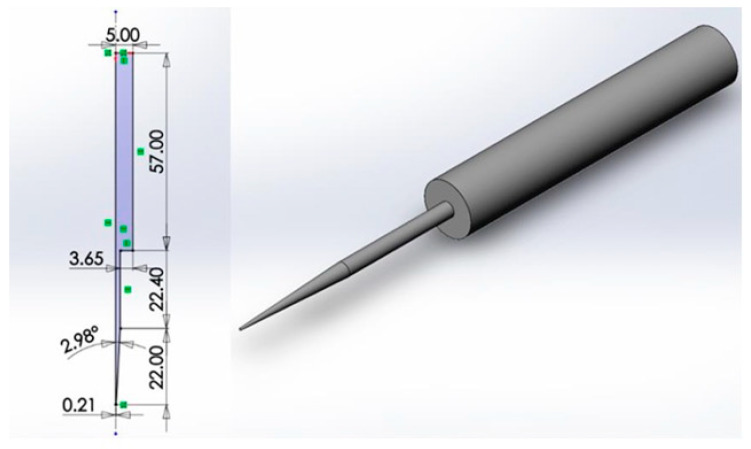
Base nozzle 3D geometry (dimensions in mm).

Computational fluid dynamics was used to model the hypothetical amount of shear stress the cells experience during the 3D printing process at the pressure and nozzle size used for 3D printing. The nozzles and syringes used in the 3D bioprinting process were created in SolidWorks (Version 2020, Vélizy-Villacoublay, France) (Figure 1) (Dassault Systèmes SE, Waltham, MA, USA); subsequently it was exported as a .stl file to TetGen software (Version 1.6.0, Berlin, Germany) to undergo tetrahedral meshing (with 2601 nodes, 464 Tet15 elements) for modelling purposes in FEBio software (Version 2.9, University of Utah, Salt Lake City, UT, USA). The boundary conditions of the internal surface of the nozzle were set as non-slip walls with the outlet pressure set at atmospheric values (zero fluid dilatation). A range of hypothetical extrusion pressures from 20 to 40 kPa were used to model the fluid velocity and shear stress within the bioink were they to be printed at these printing pressures, plotted against increasing extrusion time. The range of values was selected based on the minimum printing pressure needed to attain continuous bioink flow (30 mmHg). The shear stress was modelled as the bioink flowed through the syringe and subsequently the narrower printer nozzle over the duration of the printing process.

As the bioink exhibits shear-thinning behaviour according to many previous studies, the Carreau model (Equation (1)) was used for modelling the rheological properties of bioink. The parameters were determined by curve fitting using our previously published data on the bioink viscosity and rheology [21]:

(1) $$\mu =\mu_{\infty }+\left(\mu_{\infty }-\mu_{0}\right)\cdot {\left[1+{\left(\lambda \dot{\gamma }\right)}^{2}\right]}^{\frac{n-1}{2}}$$

where, *µ* is the dynamic viscosity of bioink; *µ_∞_* is the viscosity at infinite shear rate (*µ_∞_* = 0.00116638 Pa∙s); *µ*_0_ is the viscosity at zero shear rate (*µ*_0_ = 0.12274 Pa∙s); $\dot{\gamma }$ is the shear rate; and λ is the characteristic time (λ = 13 s).

### 2.9. Statistical Analysis

The data presented represent the mean and standard deviation of at least three technical repeats per condition, and as per Section 2.1 are derived from biological triplicates (three separate patients). Statistical comparisons were made using a two-way analysis of variance with Tukey’s post-hoc multiple comparison test. An unpaired *t*-test was used to compare the means of the LDH assay.

## 3. Results

### 3.1. Computational Modelling of Bioink Velocity and Shear Stress

**Figure 2:**
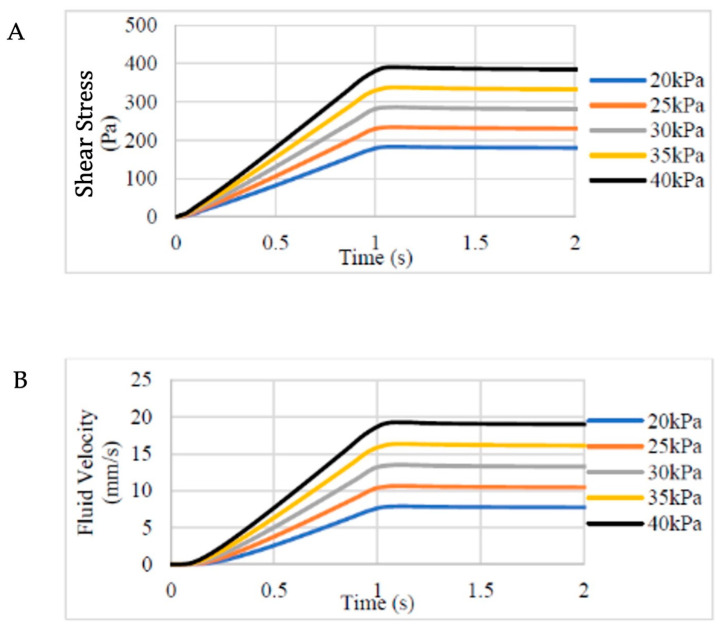
Computational modelling of fluid shear stress and velocity during the extrusion bioprinting process. (**A**) Shear fluid stress (in Pa) as a product of inlet pressure variation (kPa) is presented temporally as the ink flows through a 22 G conical nozzle over the course of 2 s, demonstrating increases in shear stress with increasing inlet pressures. (**B**) Fluid velocity (in mm/s) is expressed as a product of inlet pressure variation using a 22 G 3D printing nozzle. With increasing inlet pressures, higher levels of fluid velocity are expectedly observed within the bioink.

The computational modelling approach demonstrated that 3D bioprinting using the NCHA bioink and a range of printing pressures from 20 to 40 kPa translated to a range of shear stress (180–400 Pa) and fluid velocity (7–19 mm/s) when using the CELLINK INKREDIBLE 3D bioprinter (Figure 2). The cells within the biomaterial would be subjected to the shear stresses mentioned during their journey through the printer cartridge and conical 22 G nozzle. Of note, irrespective of printing pressure, the plateau in shear and velocity occurred at the same time point, with a period of approximately 1 s (1.08 s) until this point was achieved. In these experiments, 30 kPa was used to print the cell-laden NCHA bioink, meaning the cells experienced a maximum shear stress of 292 Pa and maximum fluid velocity of 13.4 mm/s.

### 3.2. Cell Viability Assessment

**Figure 3:**
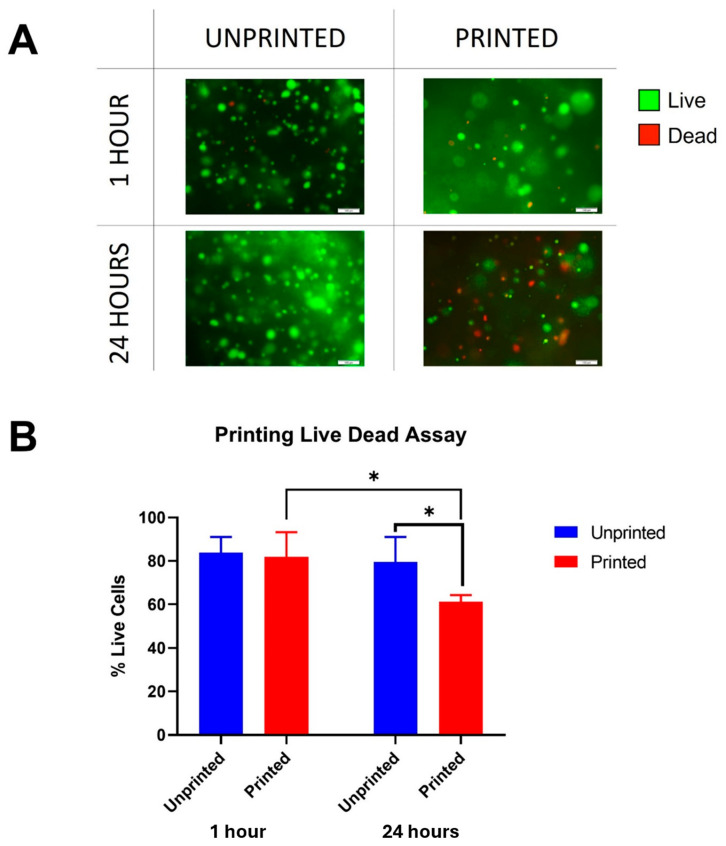
Live–dead assay of CDCs after printing at 1 h (Day 0) and 24 h (Day 1) compared to unprinted controls. (**A**) The viability of cells in 3D printed and unprinted conditions within the first hour of printing (demonstrated on the top row) appears comparable in viable cells (coloured green) at the initial timepoint, but after 24 h (bottom row), a marked increase in non-viable cells (coloured red) is evident in the printed condition. (**B**) Live–dead cell counts of viable cells as a percentage of total cells counted in three different areas of printed and unprinted crosslinked materials. The mean values are presented with pairwise comparisons from two-tailed *t*-tests. No significant differences were seen immediately post-printing but a larger amount of non-viable cells were visualised after 24 h. * *p* < 0.05.

Biologically, this translated to a reduction in cell viability as determined with the live–dead assay (Figure 3). Cells appeared largely viable in both the printed and unprinted samples at 1 h post-printing (Figure 3A), and statistically there was no immediate statistical difference in cell viability post-printing (Figure 3B). However, by 24 h, noticeable differences in the number of dead cells were noted in the printed samples (Figure 3A). Relative to the live cells, this was a significant reduction in cell viability in the printed constructs (61%) compared to the unprinted constructs (79.6%, *p* = 0.047) and the constructs tested immediately post-printing (81.9%, *p* = 0.042) (Figure 3B). This indicates that cell death from the shear stress of bioprinting may not be an immediate phenomenon, but instead a delayed one.

**Figure 4:**
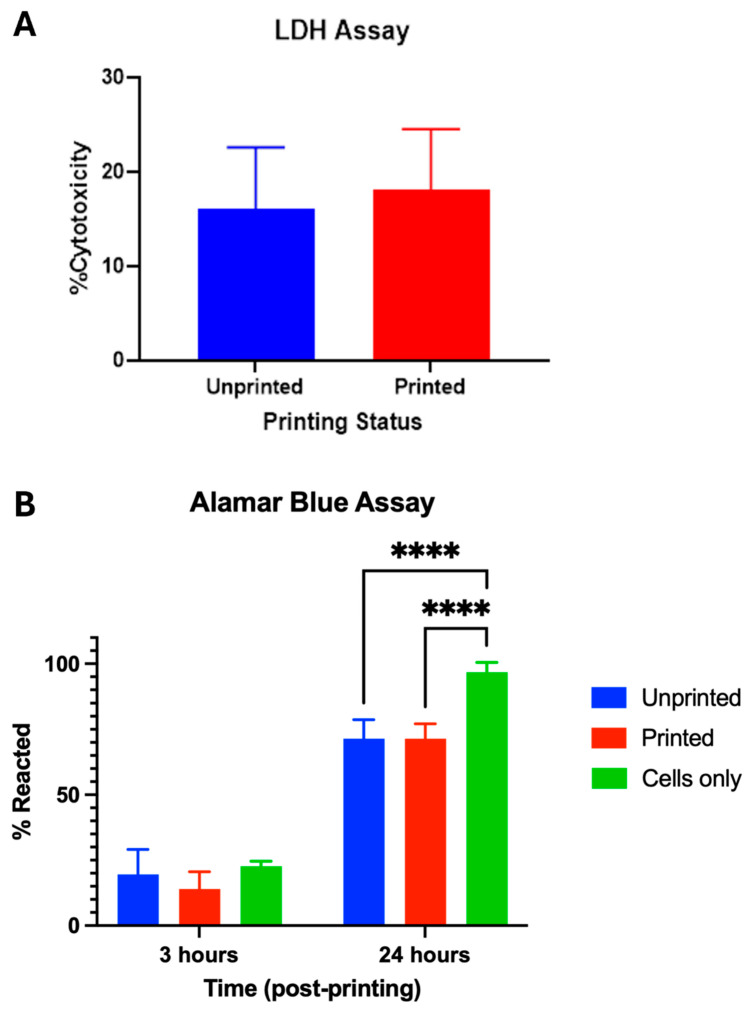
Assessments of cell lysis and metabolism in printed and unprinted cells in NCHA biomaterial. (**A**) An LDH assay of degree of cell cytotoxicity in biomaterials printed through 3D printer versus unprinted cells in biomaterial. No significant evidence of cell lysis in the printed constructs was observed immediately post-printing compared to the unprinted cells. (**B**) AlamarBlue assay of cells only, cells in biomaterial and cells in printed biomaterials at 3 h post-printing and at 24 h post-printing. **** *p* < 0.0001.

These initial findings immediately post-printing were explored further using LDH and alamarBlue assays to characterise the temporality of cell death post-printing (Figure 4). Using the LDH assay, no significant differences were seen in cell lysis between the printed and unprinted cells immediately post-printing (Figure 4A), which translated to comparable amounts of metabolic activity (*p* = 0.5) (Figure 4B) observed with the alamarBlue analysis. After 24 h of printing, there were additionally no significant differences in the metabolic activity of the cells in the printed or unprinted constructs using the alamarBlue assay (*p* = 0.9). This indicates that no biologically relevant cell lysis was evoked by the shear stress of cells and biomaterial passing through the printer nozzle at the printing pressures used in this study. Significantly less cellular metabolism was observed in both the printed and unprinted samples compared to cells only, however (*p* < 0.0001).

In summary, the combination of these assays demonstrates that there is a delayed cell death reaction post-bioprinting, which cannot be attributable to cell lysis. Our hypothesis is that this may reflect a delayed cell death pathway such as apoptosis in response to the shear stress experienced during the bioprinting process.

### 3.3. Gene Expression Analysis Following the 3D Bioprinting Process

**Figure 5:**
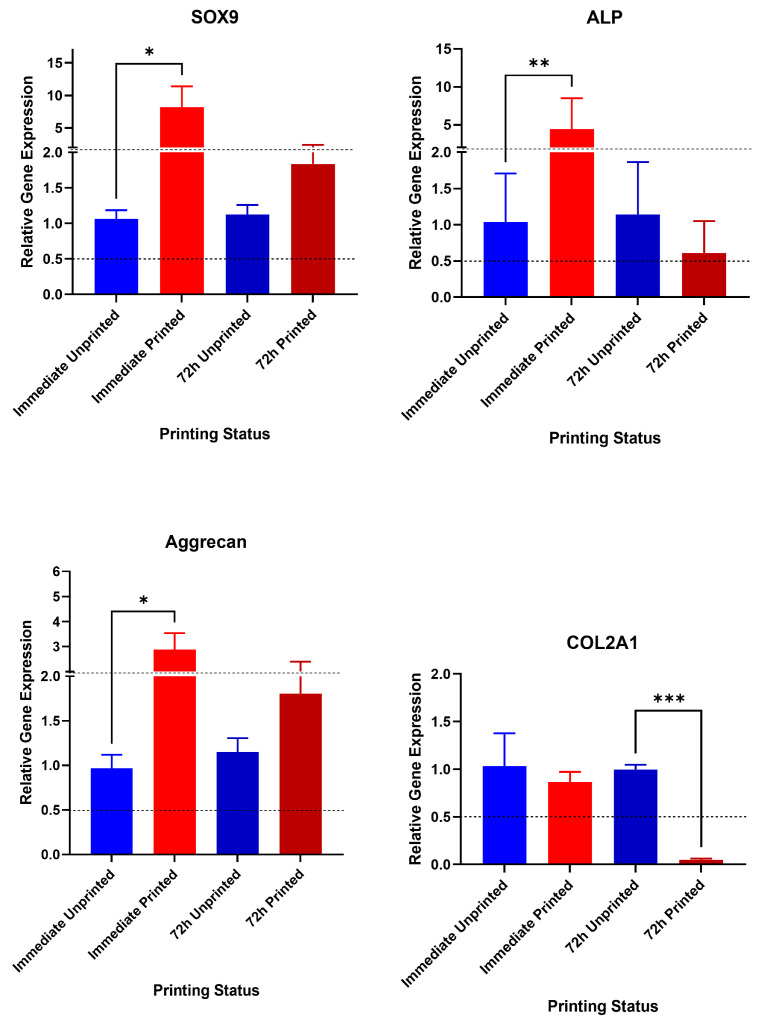
Gene expression changes immediately (4 h) after printing in NCHA bioink and 72 h after printing. Chondrogenic genes SOX9, Aggrecan and COL2A1 are presented with ALP as a marker of osteogenesis. Pairwise comparisons of the printed condition compared to the unprinted condition are presented at each timepoint with statistical significance from unpaired *t*-tests. * *p* < 0.05, ** *p* < 0.01; *** *p* < 0.001.

An additional finding of interest was in relation to gene expression changes noted as a result of the printing process (Figure 5). The printing process evoked an immediate increase in SOX9 (8.2-fold rise, *p* = 0.049), ALP (4.4-fold increase, *p* = 0.006) and aggrecan (2.9-fold increase, *p* = 0.018) detectable 4 h after printing compared to the unprinted hNSCs. However, by 72 h, there was a more comparable gene expression profile in the printed and unprinted cells (Aggrecan—*p* = 0.9; SOX9—*p* = 0.2; ALP—*p* = 0.1) with the exception of Type II collagen expression, which had fallen significantly in the printed cohort (0.05-fold change, *p* = 0.0005). Gene expression of osteocalcin and RUNX2 were also tested but were at undetectable levels in all conditions and timepoints tested. This finding indicates that both transient and delayed effects on gene expression are evoked as a result of the 3D bioprinting process, and in the instance of COL2A1 expression, this may have deleterious effects on long-term chondrogenesis.

## 4. Discussion

In this study, we sought to explore the effects of the extrusion bioprinting process on chondrocyte viability and behaviour using a combined in silico and biological approach.

Using a well-characterised nanocellulose–alginate bioink of known viscosity and rheological behaviour [21,22], we were able to apply computational modelling to calculate the shear stress within the extrusion-based bioprinting system and directly verify the effects of this process on immediate and delayed cell viability, in addition to advantageous and deleterious gene expression profiles. The printing pressures needed for extrusion of this bioink were in the region of 30 kPa when using a 22 G nozzle, which appeared to evoke levels of shear stress in the region of 292 Pa in silico. This level of shear was comparable to previously published levels of shear stress evoked by bioprinting at 50 kPa [23], but lower than previously reported biologically relevant levels of bioprinting-induced shear stress [18].

It was evident that the adverse effect on cell viability was not immediately apparent on the live–dead assay, with a stark reduction in viable cell numbers noted only after 24 h. We hypothesise that this discrepancy reflects the activation of apoptosis secondary to shear damage: a recognised pathway in response to shear [24] that occurs hours to days after injury [25]. In previous studies in vitro, exposure of chondrocytes to shear as low as 1.6 Pa over 24 h led to an increase in nitric oxide release, elevation in nucleosomal DNA fragments and reduction in BCL-2 expression: markers of apoptotic activation [26]. In fact, in our study necrosis, or cell lysis, did not appear to be a major component of the cell death pathway evoked by 3D bioprinting: the live–dead, alamarBlue and LDH assays had comparable immediate viabilities between the printed and unprinted conditions. With the information gained from this study, we acknowledge that the addition of apoptotic assays and additional time points would strengthen our understanding of the cell death mechanisms observed in this study, which we intend to exploit in further iterations of this research. Cell viability post-extrusion printing is typically reported in the region of 70–90% [12], consistent with the post-printing viability of 80% in our study; however, the 24 h viability of 65% is lower than many reported extrusion bioprinting studies, emphasising the importance of timepoint selection in providing a reflective overview of cell viability and cell death pathways. Furthermore, a delayed reduction in cell viability post-printing is a phenomenon that has been seldom reported when assessing post-bioprinting viability, with most studies reporting an increase in cell numbers within the first 72 h of printing, highlighting discrepancies between different cell types and their response to shear stress [18].

Mediating shear stress is also pivotal in ensuring lineage retention and appropriate gene expression profiles [20]. The shear stress the cells experienced in the 3D bioprinting process (roughly 290 Pa) appears to have surpassed a mechanotransductive threshold in which both chondrogenic (ACAN, SOX9) and osteogenic/hypertrophic gene expression (ALP) have been activated. In articular cartilage, high shear stress is believed to underpin osteoarthritic changes, whereas low levels of shear stress are believed to contribute to normal homeostatic mechanisms [14]. This shear stress response is mediated through the pericellular matrix, primary cilium and stretch-activated calcium channels on the surface membrane, with TGFβ1 implicated in degenerative changes [16]. The threshold of gene activation in nasoseptal and auricular cartilages is unknown, with the focus so far on understanding mechanotransduction primarily on load-bearing articular cartilage. Indeed, studies of approximately 400Pa of shear stress have demonstrated no deleterious effects on articular chondrocyte viability [27]. However, shear stress has been reported to evoke osteogenic differentiation of mesenchymal stem cells at levels as low as 1 Pa [20,28]. The gene expression effect appears to be transient with regard to bone markers, but had a latent effect on COL2 expression, which was significantly attenuated at 72 h, consistent with other studies of the biological effects of shear on chondrocytes [26]. Premature ossification and chondrocyte hypertrophy has been a concern in cartilage tissue engineering for decades, leading to construct instability, fracture and failure to emulate native tissue biomechanics, and as such we feel these effects are biologically relevant for tissue engineering efforts [27]. Efforts to downregulate chondrocyte hypertrophy, matrix mineralisation and osteogenesis must therefore be targeted not only at cell culture conditions and biomaterial selection, but must also consider printing parameters and the generation of shear stress within the bioprinting circuit, particularly when mechanically sensitive cells such as chondrocytes are required.

The biological effects of shear will differ between cell types, not only in terms of the threshold for biologically relevant signals to be evoked, but also their ability to withstand shear and preserve cellular integrity: there are some tissue types in which shear is even a desirable component of cell differentiation and motility [18]. Previous studies have explored the effect of bioprinting-induced shear stress on the viability and functionality of cells suspended in bioinks. Human umbilical vein endothelial cells printed with microvalve droplet injection bioprinting caused both short- and long-term reductions in cell viability and the network-forming capabilities of the cells post-printing [29], whereas other studies have explored the role of nozzle geometry on cell viability in the context of shear, finding shorter, conical nozzle tips to be associated with lower shear and superior viability [30,31].

We have previously demonstrated that nanocellulose-based bioinks are associated with excellent biocompatibility and cell viability, consistent with previously published literature in the bioprinting field [21,32,33]. However, further work in optimising printing parameters such as nozzle geometry and extrusion pressures is warranted: our in silico modelling predicts that even reducing the extrusion pressure by 10 kPa is expected to reduce the shear stress by approximately 1/3 (Figure 1) [34,35].

Naturally, there are a number of interventions that could be implemented to reduce the shear stress in the system that warrant further exploration, and we acknowledge the limitations of this study in not exploring different printing pressures and nozzle geometries. However, with every intervention there is a consequence for printability and potentially biocompatibility. Specifically, amending the geometry (such as the gauge) of the printer nozzle, as previously investigated, will reduce shear but at the expense of print resolution. Amending the cell type or reducing cell seeding density will also reduce shear, but will limit cell–cell interactions, extracellular matrix production and ultimately biomimicry. Reducing the viscosity of the bioink to permit lower printing pressures is an alternative solution, though rheologically, this would be incompatible with attaining prints of sufficient fidelity and resolution with conventional extrusion bioprinting. Emerging bioprinting modifications such as FRESH and SLAM bioprinting that rely on the support of a gel bath have reduced the demand on bioinks for providing shape fidelity, meaning lower viscosity bioinks can be explored and exploited as a means of promoting lower extrusion pressures and, in turn, less shear stress and superior cell viability [34,35]. Whilst this study may not have identified an optimal shear threshold for bioprinting nasoseptal chondrocytes, it highlights the nuances of managing shear stress in an extrusion bioprinting system, and the need to tailor print parameters according to biomaterial and cell type to enable durable, biomimetic constructs to be fabricated. Future work will endeavour to explore the biological and mechanical impacts of emerging printing advancements capable of printing at lower viscosities.

## 5. Conclusions

The biological relevance of shear stress is an overlooked but significant component of extrusion bioprinting with implications for cell viability and behaviour. We demonstrate that even low levels of shear stress exert biologically significant effects on human nasoseptal chondrocytes, evoking programmed cell death and reduced chondrogenic gene expression within the first 72 h post-printing using biochemical and computational methods. These findings emphasise the importance of developing low-pressure, biocompatible printing processes to augment future tissue engineering efforts and promote viable, durable and biologically relevant tissue formation. The emergence of low-viscosity, high-fidelity bioprinting methods such as SLAM and FRESH may enable the combination of low-shear, high-fidelity and high-viability printing and warrant further biocompatibility characterisation.

## 6. Patents

ISW would like to disclose a previously submitted patent application for the development of nanocellulose bioinks (Patent Pending: US16/976,803).

## Data Availability

The original contributions presented in the study are included in the article, further inquiries can be directed to the corresponding author.

## Supplementary Materials

The following supporting information can be downloaded at: https://www.mdpi.com/article/10.3390/jfb17040163/s1, Figure S1: Dimensions of 3D bioprinted hemispheres and their appearance immediately post-printing and post-crosslinking.

## References

[1] Li C., Cheung T.F., Fan V.C., Sin K.M., Wong C.W.Y., Leung G.K.K. (2017). Applications of Three-Dimensional Printing in Surgery. Surg. Innov..

[2] Jessop Z.M., Al-Himdani S., Clement M., Whitaker I.S. (2015). The Challenge for Reconstructive Surgeons in the Twenty-First Century: Manufacturing Tissue-Engineered Solutions. Front. Surg..

[3] Orlando G., Soker S., Stratta R.J., Atala A. (2013). Will Regenerative Medicine Replace Transplantation?. Cold Spring Harb. Perspect. Med..

[4] Thomas D.J., Jessop Z.M., Whitaker I.S. (2018). 3D Bioprinting for Reconstructive Surgery: Techniques and Applications.

[5] Tarassoli S.P., Jessop Z.M., Jovic T., Hawkins K., Whitaker I.S. (2021). Candidate Bioinks for Extrusion 3D Bioprinting—A Systematic Review of the Literature. Front. Bioeng. Biotechnol..

[6] Malda J., Visser J., Melchels F.P., Jüngst T., Hennink W.E., Dhert W.J.A., Groll J., Hutmacher D.W. (2013). 25th Anniversary Article: Engineering Hydrogels for Biofabrication. Adv. Mater..

[7] Jakab K., Norotte C., Damon B., Marga F., Neagu A., Besch-Williford C.L., Kachurin A., Church K.H., Park H., Mironov V. (2008). Tissue Engineering by Self-Assembly of Cells Printed into Topologically Defined Structures. Tissue Eng. Part A.

[8] Landers R., Hübner U., Schmelzeisen R., Mülhaupt R. (2002). Rapid Prototyping of Scaffolds Derived from Thermoreversible Hydrogels and Tailored for Applications in Tissue Engineering. Biomaterials.

[9] Smith C.M., Stone A.L., Parkhill R.L., Stewart R.L., Simpkins M.W., Kachurin A.M., Warren W.L., Williams S.K. (2004). Three-Dimensional BioAssembly Tool for Generating Viable Tissue-Engineered Constructs. Tissue Eng..

[10] Derakhshanfar S., Mbeleck R., Xu K., Zhang X., Zhong W., Xing M. (2018). 3D Bioprinting for Biomedical Devices and Tissue Engineering: A Review of Recent Trends and Advances. Bioact. Mater..

[11] Rutz A.L., Lewis P.L., Shah R.N. (2017). Toward Next-Generation Bioinks: Tuning Material Properties Pre- and Post-Printing to Optimize Cell Viability. MRS Bull..

[12] Millward-Sadler S.J., Salter D.M. (2004). Integrin-Dependent Signal Cascades in Chondrocyte Mechanotransduction. Ann. Biomed. Eng..

[13] Lane Smith R., Trindade M.C.D., Ikenoue T., Mohtai M., Das P., Carter D.R., Goodman S.B., Schurman D.J. (2000). Effects of Shear Stress on Articular Chondrocyte Metabolism. Biorheology.

[14] Shahin K., Doran P.M., Doran P. (2015). Shear and Compression Bioreactor for Cartilage Synthesis. Cartilage Tissue Engineering.

[15] Zhao Z.Z., Li Y., Wang M., Zhao S., Zhao Z.Z., Fang J. (2020). Mechanotransduction Pathways in the Regulation of Cartilage Chondrocyte Homoeostasis. J. Cell. Mol. Med..

[16] Gao Y., Liu S., Huang J., Guo W., Chen J., Zhang L., Zhao B., Peng J., Wang A., Wang Y. (2014). The ECM-Cell Interaction of Cartilage Extracellular Matrix on Chondrocytes. Biomed Res. Int..

[17] Jovic T.H., Zhao F., Jia H., Doak S.H., Whitaker I.S. (2024). Orbital Shaking Conditions Augment Human Nasoseptal Cartilage Formation in 3D Culture. Front. Bioeng. Biotechnol..

[18] Boularaoui S., Al Hussein G., Khan K.A., Christoforou N., Stefanini C. (2020). An Overview of Extrusion-Based Bioprinting with a Focus on Induced Shear Stress and Its Effect on Cell Viability. Bioprinting.

[19] Billiet T., Gevaert E., De Schryver T., Cornelissen M., Dubruel P. (2014). The 3D printing of gelatin methacrylamide cell-laden tissue-engineered constructs with high cell viability. Biomaterials.

[20] Yourek G., McCormick S.M., Mao J.J., Reilly G.C. (2010). Shear Stress Induces Osteogenic Differentiation of Human Mesenchymal Stem Cells. Regen. Med..

[21] Jessop Z.M., Al-Sabah A., Gao N., Kyle S., Thomas B., Badiei N., Hawkins K., Whitaker I.S. (2019). Printability of Pulp Derived Crystal, Fibril and Blend Nanocellulose-Alginate Bioinks for Extrusion 3D Bioprinting. Biofabrication.

[22] Kyle S., Jessop Z.M., Al-Sabah A., Hawkins K., Lewis A., Maffeis T., Charbonneau C., Gazze A., Francis L.W., Iakovlev M. (2018). Characterization of Pulp Derived Nanocellulose Hydrogels Using AVAP^®^ Technology. Carbohydr. Polym..

[23] Ning L., Betancourt N., Schreyer D.J., Chen X. (2018). Characterization of Cell Damage and Proliferative Ability during and after Bioprinting. ACS Biomater. Sci. Eng..

[24] Sharifi N., Gharravi A.M. (2019). Shear Bioreactors Stimulating Chondrocyte Regeneration, a Systematic Review. Inflamm. Regen..

[25] Green D.R. (2005). Apoptotic Pathways: Ten Minutes to Dead. Cell.

[26] Smith R.L., Carter D.R., Schurman D.J. (2004). Pressure and Shear Differentially Alter Human Articular Chondrocyte Metabolism: A Review. Clin. Orthop. Relat. Res..

[27] Jessop Z.M., Javed M., Otto I.A., Combellack E.J., Morgan S., Breugem C.C., Archer C.W., Khan I.M., Lineaweaver W.C., Kon M. (2016). Combining Regenerative Medicine Strategies to Provide Durable Reconstructive Options: Auricular Cartilage Tissue Engineering. Stem Cell Res. Ther..

[28] Yue D., Zhang M., Lu J., Zhou J., Bai Y., Pan J. (2019). The Rate of Fluid Shear Stress Is a Potent Regulator for the Differentiation of Mesenchymal Stem Cells. J. Cell. Physiol..

[29] Köpf M., Nasehi R., Kreimendahl F., Jockenhoevel S., Fischer H. (2022). Bioprinting-Associated Shear Stress and Hydrostatic Pressure Affect the Angiogenic Potential of Human Umbilical Vein Endothelial Cells. Int. J. Bioprint..

[30] Nair K., Gandhi M., Khalil S., Yan K.C., Marcolongo M., Barbee K., Sun W. (2009). Characterization of Cell Viability during Bioprinting Processes. Biotechnol. J..

[31] Li M., Tian X., Schreyer D.J., Chen X. (2011). Effect of Needle Geometry on Flow Rate and Cell Damage in the Dispensing-Based Biofabrication Process. Biotechnol. Prog..

[32] Markstedt K., Mantas A., Tournier I., Martínez Ávila H., Hägg D., Gatenholm P. (2015). 3D Bioprinting Human Chondrocytes with Nanocellulose-Alginate Bioink for Cartilage Tissue Engineering Applications. Biomacromolecules.

[33] Jovic T.H., Nicholson T., Arora H., Nelson K., Doak S.H., Whitaker I.S. (2023). A Comparative Analysis of Pulp-Derived Nanocelluloses for 3D Bioprinting Facial Cartilages. Carbohydr. Polym..

[34] Shiwarski D.J., Hudson A.R., Tashman J.W., Feinberg A.W. (2021). Emergence of FRESH 3D Printing as a Platform for Advanced Tissue Biofabrication. APL Bioeng..

[35] Senior J.J., Cooke M.E., Grover L.M., Smith A.M., Senior J.J., Smith A.M., Cooke M.E., Grover L.M. (2019). Fabrication of Complex Hydrogel Structures Using Suspended Layer Additive Manufacturing (SLAM). Adv. Funct. Mater..

