# Supplementary material for: Biological Impact of Extrusion Bioprinting Nasoseptal Chondrocytes for Tissue Engineering Applications
Source: J Funct Biomater. 2026 Apr 1;17(4):163. doi: 10.3390/jfb17040163 (PMC13117296; doi:10.3390/jfb17040163)
Supplement: Supplementary file 1 [file jfb-17-00163-s001.zip › jfb-4085653-supplementary.pdf]

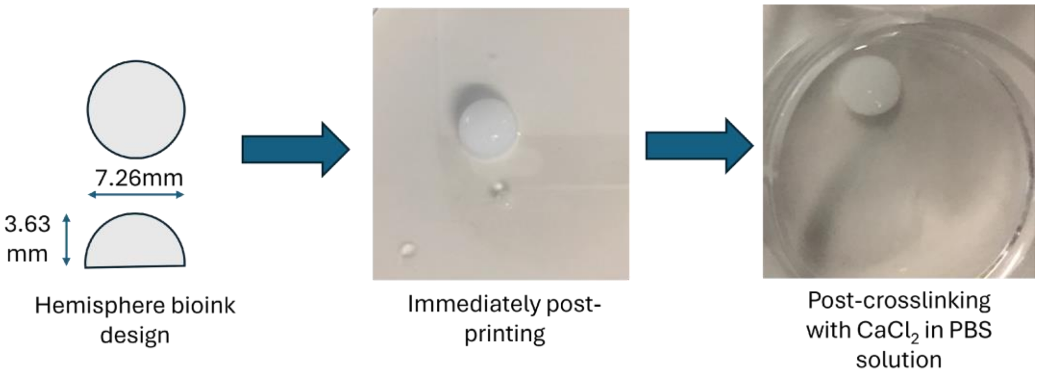

Figure S1. Dimensions of 3D bioprinted hemispheres and their appearance immediately post-printing and post-crosslinking.
